# Enlarged perivascular spaces in the basal ganglia mediate the negative impact of HbA1c levels on mild cognitive impairment

**DOI:** 10.3389/fnhum.2025.1673301

**Published:** 2025-10-20

**Authors:** Cuicui Liu, Wanhu Liu, Yimeng Yang, Yuzhu Xu, Wenjun Li, Jinyang Wang, Huiling Ren, Junyan Liu

**Affiliations:** ^1^Department of Neurology, Hebei Medical University Third Hospital, Shijiazhuang, Hebei, China; ^2^Department of Neurology, Peking University People’s Hospital, Beijing, China; ^3^Hebei Medical University Third Hospital, Shijiazhuang, Hebei, China

**Keywords:** enlarged perivascular spaces, cerebral small vessel disease, glycated hemoglobin, diabetes mellitus, mild cognitive impairment

## Abstract

**Background:**

This study aimed to investigate the mediating effect of enlarged perivascular space (EPVS) in basal ganglia (BG) on the relationship between glycated hemoglobin (HbA1c) levels and mild cognitive impairment (MCI) in patients with cerebral small vessel disease (CSVD).

**Methods:**

Data on HbA1c levels and MOCA scores and CSVD imaging markers, including EPVS volume and distribution patterns were collected. Logistic regression was performed to identify independent risk factors for MCI. A mediation effect analysis was further conducted to determine whether BG-EPVS mediate the impact of HbA1c on cognitive impairment.

**Results:**

A total of 244 CSVD patients were enrolled in this study. Compared with non-DM patients, DM patients had a significantly greater BG-EPVS volume (*p* < 0.001) and more severe periventricular white matter hyperintensities (p-WMH) (*p* = 0.036). Multivariate logistic regression analysis revealed that hypertension [odds ratio (OR) = 3.823; 95% confidence interval (CI):1.707–8.566; *p* = 0.001], the HbA1c level (OR = 1.689; 95%CI:1.255–2.272; *p*<0.001) and BG-EPVS volume (OR = 1.001; 95% CI:1.000–1.003; *p* = 0.038) were independent risk factors for MCI. After adjusting for sex and age, partial correlation analysis revealed a significant positive correlation between BG-EPVS volume and HbA1c (*β* = 0.137; *p* = 0.042) and a significant negative correlation with MOCA scores (*β* = −0.160; *p* = 0.013). The effect of HbA1c on MCI in patients with CSVD was indirectly mediated by BG-EPVS volume (indirect effect = −0.074; 95% CI: −0.187 to −0.012; the mediating effect ratio was 11.3%).

**Conclusion:**

HbA1c is an independent risk factor for MCI. Increased BG-EPVS volume mediates the partial effect of HbA1c on CSVD-related cognitive dysfunction.

## 1 Introduction

Glycated hemoglobin (HbA1c), acts as a key marker for evaluating glucose intolerance, is widely used in assessment of the risk of diabetes-related complications. Recent research has shown that elevated HbA1c (≥8%) is an important risk factor for cognitive dysfunction in patients with diabetes mellitus (DM) (Xiao et al., 2025). However, the impact of HbA1c on cognition does not seem to be limited to patients with diabetes mellitus. Systematic review revealed that HbA1c was significantly associated with structural damage to the brain, including volume decline, cortical thickness and impaired functional connectivity (Soleymani et al., 2024). Consequently we hypothesize that HbA1c induced MCI correlates with changes in brain structure.

DM is an independent determinant of CSVD. Chronic hyperglycemia potentiates brain injury through three mechanisms: blood–brain barrier disruption, deposition of advanced glycation end products, and subsequent impairment of glymphatic clearance (Chen et al., 2021; Zebarth et al., 2023). Enlarged perivascular spaces (EPVS), as imaging markers of CSVD, reflect glymphatic clearance dysfunction (Hayden, 2024; Hannawi, 2024). Previous studies have demonstrated that the mechanism underlying EPVS may involve regional heterogeneity across different brain regions. Basal ganglia (BG)-EPVS are primarily distributed around precapillary arterioles, and their formation mechanisms are potentially associated with hemodynamic disruption. In contrast, centrum semiovale (CSO)-EPVS are predominantly distributed around postcapillary venules, and their underlying mechanisms are related primarily to the accumulation of metabolic products, including *β*-amyloid and tau proteins (Na et al., 2023; Oltmer et al., 2024; Rowsthorn et al., 2023). However, the distribution patterns and pathological mechanisms of EPVS in diabetic mellitus (DM) patients remain undefined. Recent studies have confirmed that an increased EPVS burden is associated with the progression of cognitive impairment (Paradise et al., 2021). However, the effect of EPVS distribution on cognition remains controversial. Clinical studies in diabetic populations have revealed that compared with cognitively normal controls, patients with MCI have a greater BG-EPV burden (Teng et al., 2022). Other studies have shown that CSO-EPVS and hippocampal EPVS may be specifically linked to memory impairment in DM patients (Zhao et al., 2022; Pan et al., 2025). Based on this, we hypothesize that whether the distribution of EPVS mediates the impact of HbA1c on cognition.

Therefore, this study aimed to analyze the mediating effect of EPVS on the relationship between HbA1c and MCI in patients with CSVD to elucidate the mechanism of the metabolic-vascular-glycemic-like network in CSVD-related cognitive impairment and provide a theoretical basis for the development of precise intervention strategies targeting MCI in patients with DM.

## 2 Methods

### 2.1 Study design and population

This retrospective study consecutively enrolled patients diagnosed with CSVD in the Department of Neurology at the Hebei Medical University Third Hospital between July 2023 and February 2025. The inclusion criteria were as follows: (1) the diagnosis of CSVD conformed to the Standards for Reporting Vascular Changes on Neuroimaging (STRIVE) criteria (Wardlaw et al., 2013); and (2) age was ≥60 years. The exclusion criteria were as follows: (1) secondary cognitive impairment due to alcohol consumption, substance abuse, vitamin B12 deficiency, or hypothyroidism; (2) secondary cognitive impairment from neurodegenerative diseases, acute cerebral infarction, or cerebral amyloid angiopathy; and (3) inability to undergo magnetic resonance imaging (MRI) or the MoCA assessment. The study was approved by the Ethics Committee of the Hebei Medical University Third Hospital (Approval No. W2024-065-1). All patients signed the informed consent form.

### 2.2 Baseline data collection

Demographic data, including sex, age, weight, and height, and medical history, including hypertension, coronary heart disease, DM, stroke, and smoking/alcohol consumption history, were collected from medical records. Blood analyses were performed for high-density lipoprotein (HDL), total cholesterol (TC), triglyceride (TG), low-density lipoprotein (LDL), homocysteine (Hcy), glycated hemoglobin (HbA1c), and fasting blood glucose (FBG) levels. The diagnosis of diabetes was based on the diagnostic criteria of the World Health Organization (Alberti and Zimmet, 1998).

### 2.3 Neuropsychological assessment

MCI assessments were performed by two standardized trained clinicians using the Beijing Version of MoCA (Hong et al., 2022). MCI was identified as a MoCA score <26 (an additional 1 point for education <12 years) (Lu et al., 2011).

### 2.4 Imaging

Cerebral imaging was performed utilizing a 3.0 Tesla MRI system (Philips, Netherlands). The scanning protocols included three-dimensional (3D) magnetization-prepared rapid gradient-echo (MPRAGE) sequences for T1-weighted imaging (T1WI), T2-weighted imaging (T2WI), 3D fluid-attenuated inversion recovery (T2-FLAIR) sequences, diffusion-weighted imaging (DWI), and susceptibility-weighted imaging (SWI). Parameters for the 3D T1WI sequence were as follows: (repetition time/echo time = 500 ms/20 ms, flip angle = 8°, image resolution = 1 mm × 1.2 mm × 1 mm). 3D T2WI was performed with the following parameter settings: TR = 3,000 ms, TE = 90 ms, a flip angle of 90°, an image resolution maintained at 1 repetition time (TR) set to 500 ms, an echo time (TE) of 20 ms, a flip angle of 8°, and an image size of 1 mm × 1 mm × 1 mm. The parameters for 3D T2-FLAIR imaging were as follows: TR = 7,000 ms, TE = 120 ms, a flip angle of 90°, and an image resolution consistent with that of T2WI (1 mm × 1 mm × 1 mm). The DWI scanning parameters were as follows: TR = 5,000 ms, TE was set to the shortest, b0 and b1000 were used, and the resolution was 1.4 mm × 2.0 mm × 6 mm. SWI was conducted as follows: TR = 31 ms, TE = 7.2 ms, field of view of 230 mm, and V oxide size of 0.6 mm × 0.6 mm × 2.0 mm.

Assessment of CSVD imaging markers: Two radiologists reassessed neuroimaging markers, including white matter hyperintensities (WMH), lacunes, cerebral microbleeds (CMBs), and enlarged perivascular spaces (EPVS), in the original imaging data. When they disagreed, a consensus was reached through discussion. EPVS were defined as spaces with a diameter >2 mm and a signal intensity consistent with that of cerebrospinal fluid, which showed hyperintense signals on T2-weighted images and hypointense signals on T2-FLAIR images. EPVS were segmented and measured with a deep learning-based segmentation approach—the EPVS Automatic Segmentation System (VB-Net) (Zhang et al., 2024). The revised Fazekas score was used for white matter hyperintensity (WMH) assessment (Zheng et al., 2023). To ensure assessment consistency, two radiologists were blinded to the diagnosis of the patients. When they disagreed, a consensus was reached through discussion.

### 2.5 Statistical analysis

First, participants were grouped by the presence of diabetes mellitus to analyze baseline characteristics and EPVS distribution patterns among diabetic patients. Second, participants were stratified by the presence of MCI to compare baseline and imaging parameters between groups. In the univariate regression analysis for MCI risk factors, the Benjamini–Hochberg method was applied for FDR correction to control false positive risk, and variables with an adjusted *p*-value < 0.05 were included in the multivariate logistic regression model. Partial correlation analysis, controlling for age and sex, was performed in the MCI group to explore the relationships between HbA1c levels and BG-EPVS severity, as well as between BG-EPVS severity and the MoCA score. For the mediation analysis, the PROCESS toolbox was applied to investigate the mediating role of BG-EPVS in the association between HbA1c and MCI in patients with CSVD. A multivariate linear regression model was employed with model number 4 selected; 5,000 bootstrap samples were included, and bootstrap inference was selected for the model coefficients. A *p-*value ≤0.05 was considered to indicate statistical significance.

## 3 Results

### 3.1 Participant characteristics between patients with DM and those without DM

This retrospective study included 244 eligible CSVD patients, there were 134 patients with MCI, 110 patients without MCI. All enrolled patients were divided into non-DM group (*n* = 133) and a DM group (*n* = 111). Table 1 shows the differences between the two groups. Patients with DM had a greater incidence of coronary heart disease and stroke (*p* < 0.05, Table 1). The DM group also had significantly higher levels of FBG and HbA1c than the non-DM group did (*p* < 0.05, Table 1). In terms of CSVD imaging markers, DM patients presented more severe periventricular WMH and a larger volume of BG-EPVS, with statistically significant differences (*p* < 0.05, Table 1).

**Table 1:** Comparison of baseline data between the diabetes and nondiabetes groups.

Variables	non-DM group (*n* = 133)	DM group (*n* = 111)	Statistic	*p-*value
Age, years	68.91 ± 7.00	69.96 ± 7.63	1.124	0.262
Male, *N* (%)	79 (54.5)	66 (59.5)	0.000	0.992
MCI, *N* (%)	62 (46.3)	72 (53.7)	8.138	0.004^*^
Hypertension, *n* (%)	98 (73.7)	86 (77.4)	0.662	0.416
History of coronary, *n* (%)	23 (17.3)	35 (31.5)	6.769	0.009^*^
History of stroke, *n* (%)	42 (31.6)	58 (52.3)	10.691	0.001^*^
Smoking, *n* (%)	37 (27.8)	28 (25.2)	0.208	0.648
Drinking, *n* (%)	28 (21.1)	20 (18.0)	0.353	0.553
TC, mean ± SD, mmol/L	4.34 ± 1.09	4.11 ± 1.18	−1.588	0.114
TG, median (IQR), mmol/L	1.19 (0.88, 1.65)	1.34 (1.02, 1.81)	−0.622	0.534
HDL, median (IQR), mmol/L	1.16 (1, 1.4)	1.13 (0.98, 1.35)	−1.812	0.07
LDL, mean ± SD, mmol/L	2.65 ± 0.83	2.50 ± 0.98	−1.298	0.196
FBG, median (IQR), mmol/L	5.55 (4.94,6.27)	7.48 (6.5, 9.06)	−2.164	0.03^*^
HCY, median (IQR), mmol/L	14.68 (12.26,16.17)	13,21 (10.22, 16.9)	−1.165	0.244
HbA1c, median (IQR), %	5.7 (5.48, 6.1)	6.95 (6.3,8.18)	−4.194	<0.001^*^
Imaging markers of CSVD				
pWMH (point)			6.651	0.036^*^
1	45 (33.9)	25 (22.5)		
2	62 (46.6)	70 (63.1)		
3	26 (19.5)	16 (14.4)		
dWMH (point)			0.314	0.855
1	57 (42.9)	45 (40.5)		
2	54 (40.6)	49 (44.1)		
3	22 (16.5)	17 (15.0)		
No. of lobar CMBs, *n* (%)	0 (0, 1)	0 (0, 2)	−0.459	0.646
No. of lacunes, *n* (%)	0 (0, 2)	1 (0, 2)	−1.518	0.129
Volume of BG-EPVS, median (IQR), mm^3^	426.44 (320.65, 545.06)	525.48 (366.20, 698.94)	−4.168	<0.001^*^
Volume of CSO-EPVS, median (IQR), mm^3^	48.53 (17.05,84.89)	55.70 (25.42, 98.41)	−1.798	0.072

*Denotes significance at a *p-*value of <0.05.

### 3.2 Logistic regression analysis of risk factors for MCI in patients with CSVD

Univariate and multivariate logistic regressions were performed to identify risk factors for MCI in CSVD patients, and the results are shown in Table 2. Univariate logistic analysis revealed that age, hypertension, History of HbA1c, pWMH, dWMH, BG-EPVS volume, and centrum semiovale EPVS volume were significantly associated with MCI (adjusted *p* < 0.05, Table 2). After adjusting for confounding factors, multivariate logistic regression analysis revealed that hypertension [odds ratio (OR): 3.823, 95% confidence interval (CI): 1.707–8.566, *p* = 0.001], HbA1c levels (OR: 1.689, 95% CI: 1.255–2.272, *p*<0.001), and BG-EPVS volume (OR: 1.001, 95% CI: 1.000–1.003, *p* = 0.038) were independently associated with MCI (Table 2).

**Table 2:** Univariate and multivariate logistic regression analyses of factors associated with MCI.

Variable	Univariate logistic analysis		Multivariate logistic analysis	
	OR (95% CI)	Adjusted *p-*value	OR (95% CI)	*p-*value
Male	1.216 (0.654–2.26)	0.705		
Age	1.049 (1.012–1.087)	0.027	1.021 (0.977–1.067)	0.35
BMI	0.977 (0.897–1.065)	0.741		
Hypertension	3.67 (1.915–7.035)	0.021	3.823 (1.707–8.566)	0.001^*^
History of coronary disease	1.735 (0.901–3.34)	0.061		
History of stroke	2.005 (1.174–3.597)	0.032	1.605 (0.85–3.03)	0.144
Smoking	1.185 (0.542–2.588)	0.705		
Drinking	0.632 (0.27–1.48)	0.41		
TC	0.858 (0.684–1.078)	0.282		
TG	1.063 (0.818–1.381)	0.757		
HDL	0.376 (0.155–0.911)	0.063		
LDL	0.939 (0.707–1.248)	0.735		
FBG	1.123 (1.015–1.243)	0.058		
HCY	1.002 (0.965–1.041)	0.906		
HbA1c	1.744 (1.329–2.288)	0.011	1.689 (1.255–2.272)	<0.001^*^
pWMH	2.426 (1.601–3.676)	0.007	1.692 (0.778–3.678)	0.185
dWMH	2.017 (1.381–2.945)	0.005	1.017 (0.48–2.156)	0.964
No. of lobar CMBs	1.095 (0.997–1.204)	0.21		
No. of lacunes	1.106 (0.970–1.261)	0.212		
Volume of BG-EPVS	1.002 (1.001–1.003)	0.004	1.001 (1.000–1.003)	0.038^*^
Volume of CSO-EPVS	1.005 (1.001–1.010)	0.004	1.004 (0.998–1.01)	0.174

^*^Denotes significance at a *p-*value of <0.05.

All patients were divided into two subgroups for univariate and multivariate regression analyses to identify the risk factors for MCI based on the presence or absence of comorbid DM. The results showed that hypertension, HbA1c levels and BG-EPVS volume were independent predictor of MCI only in DM group (Supplementary materials 1, 2).

### 3.3 Partial correlation analysis of BG-EPVS volume with HbA1c levels and the MoCA score

**Figure 1:**
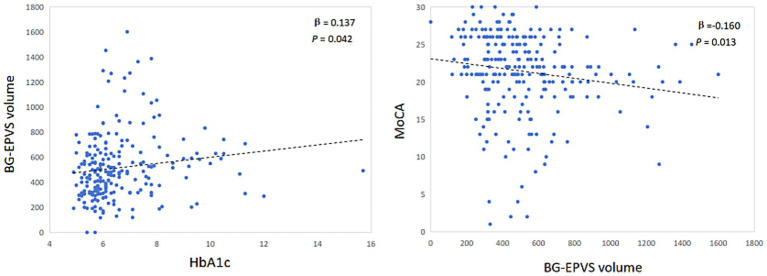
Partial correlation analysis of BG-EPVS volume and HbA1c, MoCA.

After controlling for sex, age, hypertension and history of stroke, the BG-EPVS volume was significantly correlated with HbA1c levels (*β* = 0.137, *p* = 0.042) and significantly negatively correlated with the MoCA score (*β* = −0.160, *p* = 0.013) (Figure 1).

### 3.4 Indirect effects of HbA1c levels on MCI mediated by BG-EPVS volume

**Figure 2:**
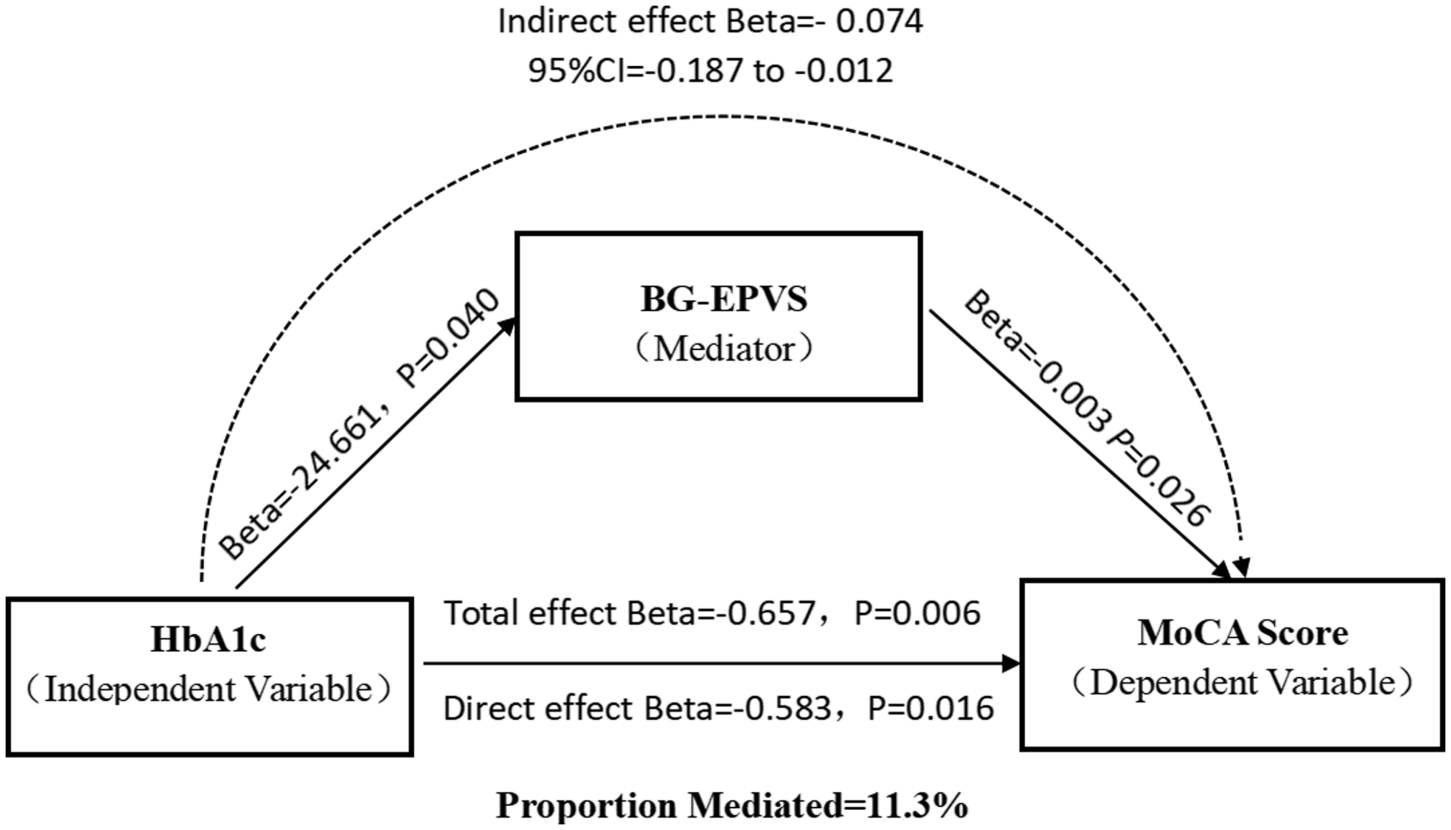
Mediation effect diagram. Association of HbA1c with basal ganglia EPVS volume. Association of basal ganglia EPVS volume with MoCA score. Total effect of HbA1c on MoCA score. Indirect effect of HbA1c on MoCA score mediated by BG-EPVS volume. Direct effect of HbA1c on MoCA score.

Mediation analysis revealed significant natural indirect (*β* = −0.074, 95%CI: −0.187 to −0.012), direct (*β* = −0.583, *p* = 0.016), or total effects (*β* = −0.657, *p* = 0.006) of HbA1c levels on cognitive impairment that were mediated by BG-EPVS volume in patients with CSVD, with a 11.3% mediating effect (Figure 2). However, the aforementioned mediation effect does not hold in the DM group (Supplementary material 3).

## 4 Discussion

MCI is a crucial clinical manifestation of CSVD and is a significant risk factor for subsequent decreases in activities of daily living and an increased fall risk (Zhou et al., 2024). T2DM is a significant risk factor for both CSVD and MCI. Recent studies have indicated that the prevalence of MCI in individuals with T2DM ranges from 30.7% to 44.1% (Yu and Yu, 2025; Zhao et al., 2025). Our study investigated the role of CSVD imaging markers in the pathway through which glycemic metabolic indicators influence cognitive function. The findings revealed that the HbA1c level and BG-EPVS volume were independent risk factors for cognitive impairment in patients with CSVD, but the association was not significant in patients without DM. Moreover, the BG-EPVS volume was positively correlated with the HbA1c level (*β* = 0.137; *p* = 0.042) and significantly negatively correlated with the MoCA score (*β* = −0.160; *p* = 0.013). In all patients, mediation analysis indicated that the BG-EPVS volume partially mediated the negative effect of the HbA1c level on the MoCA score, supporting the role of CSVD as a pathway through which metabolic dysfunction affects brain health. However, when stratifying patients by presence or absence of DM, this mediation effect disappeared.

This study demonstrated that the HbA1c level serves as an independent predictor of MCI in patients with CSVD, especially among those with DM. Specifically, every 1% increase in HbA1c was associated with a 77.7% increase in the risk of MCI development, which aligns with previous findings indicating that every 1% increase in HbA1c was associated with a 0.021-point decrease in cognitive screening assessment scores (Cukierman-Yaffe et al., 2021). Importantly, these findings demonstrate population-level generalizability and are independent of ethnicity. Epidemiological research among Asian Americans in the United States has demonstrated that elevated HbA1c levels are linked to declines in executive cognitive function and an increased incidence of MCI (OR = 1.20; 95% CI: 1.11–1.29) (Gonzalez et al., 2024). Studies of Latino communities have also increasingly recognized an association between the HbA1c level and cognitive impairment (Yu and Siang Ng, 2023). The quantitative threshold of the effect of HbA1c on cognition is generally consistent across studies. A meta-analysis of Chinese DM patients indicated that an HbA1c level ≥ 8.5% is an independent predictor of cognitive dysfunction (Peng et al., 2023). Another study revealed a U-shaped association of mean HbA1c with cognitive dysfunction, where HbA1c levels ≥8% conferred a greater risk than stable ranges did (Xiao et al., 2025). Notably, elevated HbA1c levels remain significantly associated with cognitive impairment even in studies focusing on coronary heart disease (Zheng et al., 2025).

EPVS represent imaging manifestations of impaired intracranial lymphatic circulation and are most commonly observed in the BG and CSO regions. Our research revealed that compared with non-T2DM individuals, T2DM patients presented larger BG-EPVS volume, whereas no significant difference in CSO-EPVS volume was detected between the two groups. Partial correlation analysis in CSVD patients confirmed a dose-dependent association between HbA1c levels and BG-EPVS severity, reinforcing the link between glycemic control and BG-EPVS formation. Studies by Zou et al. (2022) and Bown et al. (2023) have indicated that cerebrovascular risk factors, including hypertension and diabetes, significantly contribute to the severity of Choi et al. (2023) BG-EPVS. Choi et al. (2023) demonstrated that type 2 diabetes mellitus (T2DM) patients exhibit progressive basal ganglia volume reduction, which is correlated with gray matter atrophy in cortical-striatal-limbic circuits during disease progression. Notably, studies in nondiabetic older adults have demonstrated an association between BG-EPVS formation and insulin resistance (Wu et al., 2020), whereas no such correlation was observed for CSO-EPVS. These findings collectively demonstrate that the basal ganglia are a selectively vulnerable target of dysglycemia-induced neurotoxicity and that dysglycemia may selectively modulate EPVS in the BG region.

This study also revealed that increased BG-EPVS volume was significantly associated with MCI and served as an independent risk factor for MCI in CSVD patients. However, no significant correlation was detected between CSO-EPVS volume and MCI. Teng et al. (2022) demonstrated that cognitive decline in T2DM patients was associated with BG-EPVS formation (OR = 3.84; 95% CI: 1.81–8.13; *p* < 0.001), and as the severity of the BG-EPVS increased, the MoCA score significantly decreased. Another study similarly suggested that BG-EPVS severity might serve as a potential imaging marker for cognitive impairment in T2DM patients, outperforming CSO-EPVS (Choi et al., 2021), which aligns with our findings. However, Choe et al. (2022) reported that BG-EPVS were associated with only executive dysfunction, and this correlation weakened after adjusting for lacunar infarcts and WMH, indicating that the influence of BG-EPVS on executive function may be modulated by other CSVD imaging markers.

Our study further indicated that BG-EPVS partially mediated the effect of HbA1c levels on MCI in patients with CSVD. Elevated HbA1c levels promote the progression of CSVD, and this pathway contributes to the exacerbation of cognitive decline. In animal models of DM, reduced glymphatic transport and impaired cerebrospinal fluid–interstitial fluid exchange have been observed, suggesting that DM leads to dysfunction of neurovascular–glial complexes, which contributes to EPVS formation (Benveniste and Nedergaard, 2022). HbA1c levels specifically regulate EPVS distribution, with BG-EPVS contributing to HbA1c-induced MCI through the following mechanisms: (1) BG-EPVS primarily localize around precapillary microarteries (Na et al., 2023), and their formation is linked to atherosclerotic/hemodynamic injury and drives vascular cognitive impairment (Zhong et al., 2023; Yamasaki et al., 2022). The indirect effect of hyperglycemia on MCI via BG-EPVS suggests a mechanism involving cerebral hemodynamic injury, thereby illuminating the role of the “metabolism–vascular–glymphatic” interactive network in CSVD-related MCI. In contrast, CSO-EPVS predominantly localize around postcapillary venules (Na et al., 2023). Their pathogenesis is associated primarily with *β*-amyloid and tau protein deposition, which is a critical factor in Alzheimer’s disease-related dementia (Perosa et al., 2022). However, notably, the effect of HbA1c on the MoCA score in DM patients is not mediated by BG-EPVS volume. This negative result stems from the absence of a significant correlation between HbA1c and BG-EPVS, suggesting that the development of BG-EPVS in patients with DM is not dependent on HbA1c levels. Instead, the development of BG-EPVS in patients with DM may be influenced by other cerebrovascular risk factors. This speculation is supported by our finding that compared with the non-DM group, the DM group was more likely to have comorbid cerebrovascular disease risk factors.

The main strength of our study is that we analyzed the impact of HbA1c levels on both BG-EPVS and cognitive function and further investigated the role of increased BG-EPVS volume in the association between elevated HbA1c levels and cognitive impairment. This approach helps elucidate the potential pathogenic mechanisms underlying cognitive dysfunction in diabetic patients. However, several limitations should be acknowledged. First, as this was a single-center study with a relatively small sample size, selection bias may have affected the results. Second, the potential confounding effects of glucose-lowering medications on the outcomes were not considered. Additionally, the MoCA score is significantly influenced by educational level, resulting in a lack of a unified cutoff value for diagnosing MCI using the MoCA. In this study, only two stratifications were made on the basis of years of education during the MCI identification process, which may introduce bias into MCI diagnosis on the basis of MoCA scores. In the future, stratified studies of the educational background of the population and multicenter large-scale studies are warranted to address these limitations and validate our findings.

## 5 Conclusion

In summary, our findings demonstrate that elevated HbA1c levels are associated with increased BG-EPVS volumes and MCI. Furthermore, the partial effect of HbA1c elevation on cognitive impairment may be mediated by the BG-EPVS volume, which provides novel insights into the pathogenic mechanisms underlying cognitive impairment in diabetic patients. These findings highlight BG-EPVS as a potential neuroimaging marker for assessing the risk of MCI in patients with SCVD.

## Data Availability

The raw data supporting the conclusions of this article will be made available by the authors, without undue reservation.
