# Supplementary material for: Enlarged perivascular spaces in the basal ganglia mediate the negative impact of HbA1c levels on mild cognitive impairment
Source: Front Hum Neurosci. 2025 Oct 20;19:1673301. doi: 10.3389/fnhum.2025.1673301 (PMC12580363; doi:10.3389/fnhum.2025.1673301)
Supplement: Supplementary file 1 [file Data_Sheet_1.docx]

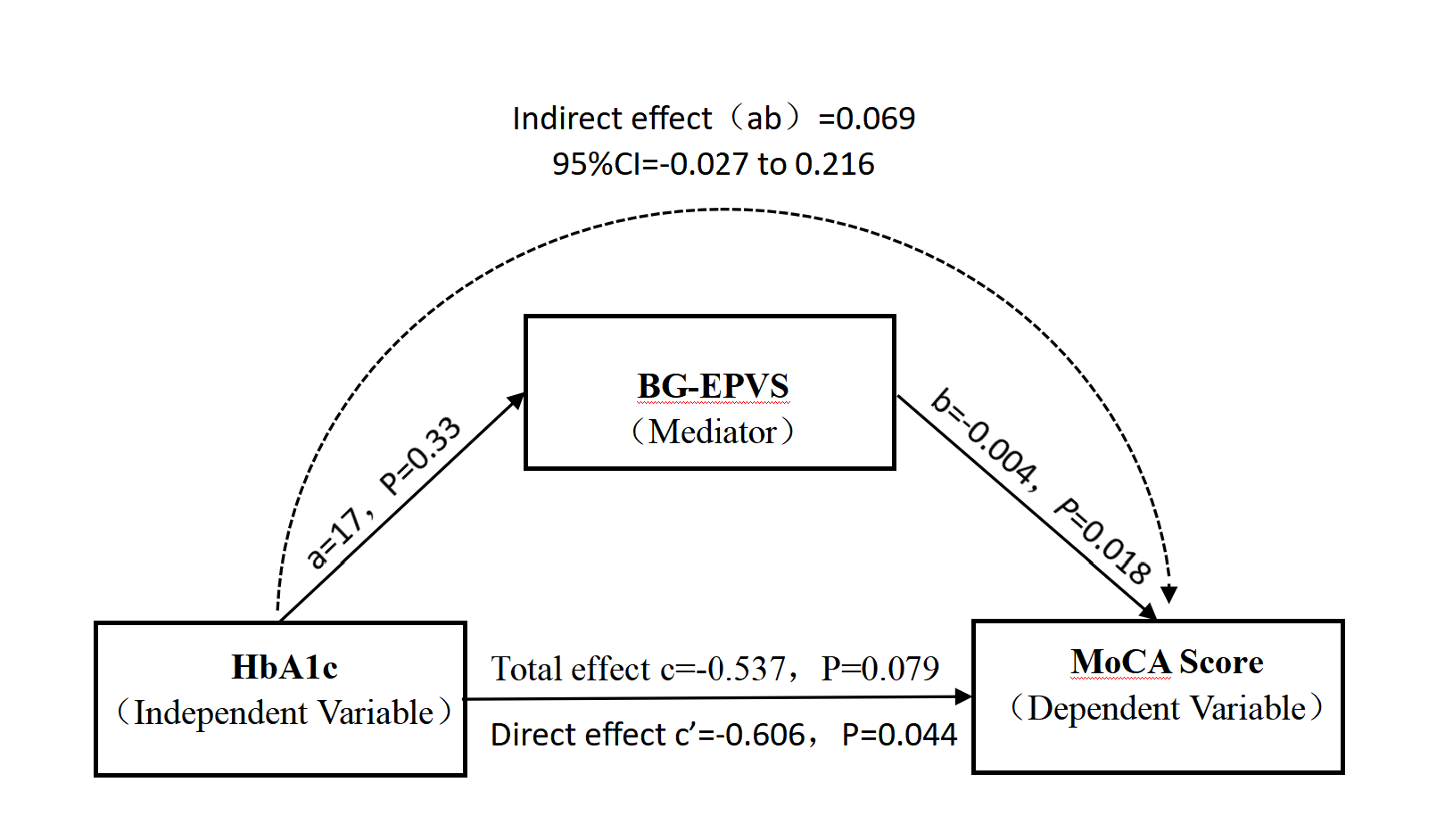


Supplementary Material 3 Mediation effect diagram in patients with DM

**Note**: **a**: Association of HbA1c with basal ganglia EPVS volume. **b**: Association of basal ganglia EPVS volume with MoCA score. **c**: Total effect of HbA1c on MoCA score. **ab**: Indirect effect of HbA1c on MoCA score mediated by BG-EPVS volume. **c'**: Direct effect of HbA1c on MoCA score.
