# Supplementary material for: Enlarged perivascular spaces in the basal ganglia mediate the negative impact of HbA1c levels on mild cognitive impairment
Source: Front Hum Neurosci. 2025 Oct 20;19:1673301. doi: 10.3389/fnhum.2025.1673301 (PMC12580363; doi:10.3389/fnhum.2025.1673301)
Supplement: Supplementary file 2 [file Table_1.docx]

Supplementary Material 1 Univariate and multivariate logistic regression analyses of factors associated with MCI in patients with DM

| Variable | Univariate logistic analysis | | Multivariate logistic analysis | |
| --- | --- | --- | --- | --- |
|  | OR(95% CI) | Adjusted P value | OR(95% CI) | *P* value |
| Male | 1.031(0.467-2.28) | 0.939 |  |  |
| age | 1.026(0.975-1.08) | 0.327 |  |  |
| BMI | 0.963(0.853-1.086) | 0.535 |  |  |
| Hypertension | 2.727(1.081-6.881) | 0.034 | 3.734(1.133-12.305) | 0.03^*^ |
| History of coronary disease | 1.884(0.776-4.573) | 0.161 |  |  |
| History of stroke | 2.372(1.067-5.273) | 0.034 | 4.445(1.499-13.158) | 0.007 |
| Smoking | 2,643(1.125-6,214) | 0.026 | 0.293(0.08-1.07) | 0.063 |
| Drinking | 2.75(1.025-7.379) | 0.045 | 1.554(0.364-6.647) | 0.552 |
| TC | 0.945(0.678-1.317) | 0.739 |  |  |
| TG | 1.349(0.877-2.075) | 0.172 |  |  |
| HDL | 0.268(0.7-1.021) | 0.054 |  |  |
| LDL | 1.056(0.704-1.584) | 0.791 |  |  |
| FBG | 1.094(0.967-1.237) | 0.152 |  |  |
| HCY | 1.006(0.946-1.071) | 0.84 |  |  |
| HbA1c | 1.754(1.198-2.568) | 0.004 | 2.239(1.375-3.644) | 0.001^*^ |
| pWMH | 2.181(1.092-4.354) | 0.027 | 1.42(0.629-3.203) | 0.399 |
| dWMH | 1.293(0.735-2.273) | 0.373 |  |  |
| No. of lobar CMBs | 1.296(0.958-1.754) | 0.092 |  |  |
| No. of lacunes | 1.045(0.847-1.289) | 0.682 |  |  |
| Volume of BG-EPVS | 1.003(1.001-1.004) | 0.005 | 1.003(1.000-1.005) | 0.019^*^ |
| Volume of CSO-EPVS | 1.006(0.999-1.013) | 0.084 |  |  |
| **Note:** *Denotes significance at a P value of <0.05. | | | | |
