# Supplementary material for: Enlarged perivascular spaces in the basal ganglia mediate the negative impact of HbA1c levels on mild cognitive impairment
Source: Front Hum Neurosci. 2025 Oct 20;19:1673301. doi: 10.3389/fnhum.2025.1673301 (PMC12580363; doi:10.3389/fnhum.2025.1673301)
Supplement: Supplementary file 3 [file Table_2.docx]

Supplementary Material 2 Univariate and multivariate logistic regression analyses of factors associated with MCI in patients without DM

| Variable | Univariate logistic analysis | | Multivariate logistic analysis | |
| --- | --- | --- | --- | --- |
|  | OR(95% CI) | Adjusted P value | OR(95% CI) | *P* value |
| Male | 1.492(0.741-3.002) | 0.262 |  |  |
| age | 1.067(1.013-1.124) | 0.014 | 1.051(0.992-1.113) | 0.09 |
| BMI | 0.991(0.875-1.123) | 0.887 |  |  |
| Hypertension | 5.116(2.04-12.83) | ＜0.001 | 3.959(1.474-10.53) | 0.006^*^ |
| History of coronary disease | 1.618(0.654-4.005) | 0.298 |  |  |
| History of stroke | 1.860(0.88-4.827) | 0.1 |  |  |
| Smoking | 2.016(1.74-8.919) | 0.096 |  |  |
| Drinking | 2.061(0.88-4.827) | 0.096 |  |  |
| TC | 0.825(0.595-1.143) | 0.248 |  |  |
| TG | 0.803(0.538-1.198) | 0.283 |  |  |
| HDL | 0.575(0.172-1.917) | 0.368 |  |  |
| LDL | 0.883(0.580-1.344) | 0.561 |  |  |
| FBG | 0.968(0.715-1.31) | 0.831 |  |  |
| HCY | 1.013(0.964-1.065) | 0.604 |  |  |
| HbA1c | 1.771(0.799-3.927) | 0.16 |  |  |
| pWMH | 2.542(1.502-4.301) | ＜0.001 | 0.847(0.316-2.273) | 0.742 |
| dWMH | 2.957(1.729-5.059) | ＜0.001 | 2.835(1.068-7.572) | 0.036^*^ |
| No. of lobar CMBs | 1.072(0.98-1.172) | 0.131 |  |  |
| No. of lacunes | 1.133(0.957-1.34) | 0.146 |  |  |
| Volume of BG-EPVS | 1.002(1-1.003) | 0.074 |  |  |
| Volume of CSO-EPVS | 1.004(0.997-1.010) | 0.268 |  |  |
| **Note:** *Denotes significance at a P value of <0.05 | | | | |
